# Characterization of Placental Infection by Zika Virus in Humans: A Review of the Literature

**DOI:** 10.1055/s-0040-1712126

**Published:** 2020-06-19

**Authors:** Emanuella Meneses Venceslau, José Paulo Guida, Eliana Amaral, José Luis Proença Modena, Maria Laura Costa

**Affiliations:** 1 Departament of Gynecology and Obstetrics, Universidade Estadual de Campinas, Campinas, SP, Brazil; 2 Departament of Genetics, Evolution and Bioagents, Universidade Estadual de Campinas, Campinas, SP, Brazil

**Keywords:** Zika virus, ZIKV, placenta, Hofbauer cells, TORCH, Zika vírus, ZIKV, placenta, células de Hofbauer, TORCH

## Abstract

**Objective** The aim of the current review is to present a systematic evaluation of reported human placental findings in cases of zika virus (ZIKV) infection.

**Data sources** We reviewed the EMBASE, PUBMED, and SCIELO databases until June 2019, without language restrictions.

**Selection of studies** The search terms *placenta* AND *zika virus* were used. The inclusion criteria of the studies were studies that reported placental findings in humans. Experimental studies, reviews, notes or editorials were excluded. A total of 436 studies were retrieved; after duplicate exclusion, 243 articles had their titles screened, and 128 had their abstract read; of those, 32 were included in the final analysis (18 case reports, 10 case series, and 4 cohorts)

**Data collection** We collected data concerning the author, year of publication, study design, number of participants, number of placental samples, onset of symptoms, perinatal outcomes, and main findings on histological analysis.

**Data synthesis** The placental pathologic findings were described as mild and nonspecific, similar to those of other placental infections, including chronic placentitis, chronic villitis, increased Hofbauer cells, irregular fibrin deposits, increased mononuclear cells in the villus stroma, villous immaturity, edema, hypervascularization, stromal fibrosis, calcification, and focal necrosis of syncytiotrophoblasts.

**Conclusion** Zika infection presents unspecific placental findings, similar to other infections in the toxoplasmosis, other agents, rubella, cytomegalovirus, and herpes (TORCH)group. Characterizing and standardizing placental findings after zika virus infection is key to understanding the mechanisms of congenital diseases.

## Resumo

**Objetivo** O objetivo desta revisão é apresentar uma avaliação sistemática dos achados relacionados à infecção por zika vírus (ZIKV) na placenta humana.

**Fontes de dados** As bases de dados EMBASE, PUBMED, e SCIELO foram pesquisadas, até junho de 2019, sem qualquer restrição de língua.

**Seleção dos estudos** Os termos *placenta* E *zika virus* foram utilizados na busca. Foram incluídos estudos que reportassem achados placentários de infecção em seres humanos, enquanto estudos experimentais, revisões, notas e editoriais foram excluídos. Um total de 436 estudos foram identificados, e 243 tiveram seus títulos lidos após a exclusão de duplicatas. Cento e vinte e oito artigos tiveram seus resumos avaliados, dos quais 32 foram incluídos na análise final (18 relatos de caso, 10 séries de casos, e 4 estudos de coorte).

**Dados obtidos** Foram pesquisados dados relativos ao autor, ano da publicação, desenho do estudo, número de participantes, número de amostras de placenta, início dos sintomas, desfechos perinatais, e principais achados histológicos.

**Síntese dos dados** Os principais achados placentários descritos foram leves e inespecíficos, similares a outras infecções placentárias, incluindo infecção placentária crônica, vilosite crônica, aumento das células de Hofbauer, depósitos irregulares de fibrina, aumento das células mononucleares no estroma viloso, imaturidade vilosa, edema, hipervascularização, fibrose estromal, calcificação, e necrose focal dos sincicitrofoblastos.

**Conclusão** Infecções por ZIKV têm achados placentários inespecíficos, similares aos de outras infecções do grupo toxoplasmose, rubéola, citomegalovírus e herpes (TORCH). Caracterizar e padronizar os achados placentários após infecção por ZIKV é fundamental para entender o mecanismo das infecções congênitas.

## Introduction

Zika virus (ZIKV) is a flavivirus much similar to other arboviruses of relevance, such as dengue, West Nile, yellow fever, and Japanese encephalitis viruses. It is transmitted mostly by *Aedes aegypti* mosquitoes, and was first recognized in humans in Uganda in 1952, with two main previous outbreaks, in Yap, Micronesia, in 2007 and in the French Polynesia, in 2013. 1 2 The ZIKV may also be transmitted to humans according to other routes non-vector reliant, such as blood transfusion, sexual transmission, or maternal-fetal transmission. 3

Brazil had the most significant and recent outbreak of ZIKV, with major relevance not only due to the total number of cases reported (over 200 thousand), but also because of its severity and association to fetal malformations. 4 The fetal consequences were further defined as Congenital Zika Syndrome (CZS), which includes a spectrum of congenital defects (not only microcephaly). 5 These conditions are similar of those caused by “TORCH” pathogens. The TORCH acronym stands for: ***T*** *oxoplasma gondii* infection, **O** ther ( *Treponema pallidum, Listeria monocytogenes, parvovirus* B-19, and human immunodeficiency virus (HIV), amongst others), **R** ubella, **C** ytomegalovirus (CMV), and **H** erpesviruses (HSV) 1 and 2. After the Brazilian zika outbreak, some authors have suggested the inclusion of ZIKV among the group “others” in the acronym or even a more direct inclusion such as TORCHZ. 6

The precise mechanisms of placental infection and maternal-fetal transmission during pregnancy, not only in ZIKV but in the other TORCH infections as well, remains unclear. Described routes include: ascending infection, direct crossing or infection of syncytiotrophoblasts (SYN), infection of extravillous trophoblasts through maternal microvasculature, and trafficking of and/or signaling from maternal immune cells. 6

The SYN layer is the outer layer of the placental villus, of multinucleated, terminally-differentiated cells in direct contact with the maternal blood. The extravillous trophoblasts (EVTs) anchor cells to the uterine wall. Both of these are differentiated from the cytotrophoblast layer (CTB) throughout pregnancy. 7 Hofbauer cells (HCs) are placental macrophages of fetal origin, existent in the chorionic villus throughout the entire gestation. 8 Hofbauer cells have been associated to ZIKV infection, with description of hyperplasia of such cells in the placenta. 9

The study of placentas of suspected cases of ZIKV is recommended, as part of optimum healthcare for these women and newborn. Histopathologic examination of the placenta, with ZIKV ribonucleic acid (RNA) testing (via reverse transcription-polymerase chain reaction [rRT-PCR]), may confirm fetal infection, since viral detection in the serum is time-sensitive and the window for ZIKV detection might be missed. 4

The aim of the present review is to present an integrative evaluation of reported placental findings in human studies on ZIKV infection during pregnancy.

## Methods

We performed a review of the literature to identify studies that assessed placental findings in human with ZIKV infection during pregnancy. The time end-point of this review was June 2019, including publications of the EMBASE, PUBMED, and SCIELO databases, without language restrictions. We used the following Medical Subject Heading (MeSH) search terms: *placenta* AND *zika virus* . The inclusion criterion of the studies was reporting of placental findings in humans, while studies that did not report placental findings, experimental studies, reviews, notes or editorials were excluded. The current study followed all recommendations of the Preferred Reporting Items for Systematic Reviews and Meta-Analyses (PRISMA) statement. In the first step of this review, two independent reviewers performed a title screening of all studies identified in the database search; in the second step, the remaining studies were evaluated considering their abstracts by two independent reviewers and further full text, for inclusion. Discordances between the primary reviewers were solved by a third senior reviewer. After the final selection of the studies that were included in this review, each study was evaluated, and the following characteristics for each study were obtained: author, year of publication, study design, number of participants, number of placental samples, onset of ZKV infection symptoms, perinatal outcomes, and main findings on histological analysis. Those results were stored in a Microsoft Excel spreadsheet (Microsoft Corp., Redmond, WA, USA) and further organized in a table with detailed description of data.

## Results

**Fig. 1:**
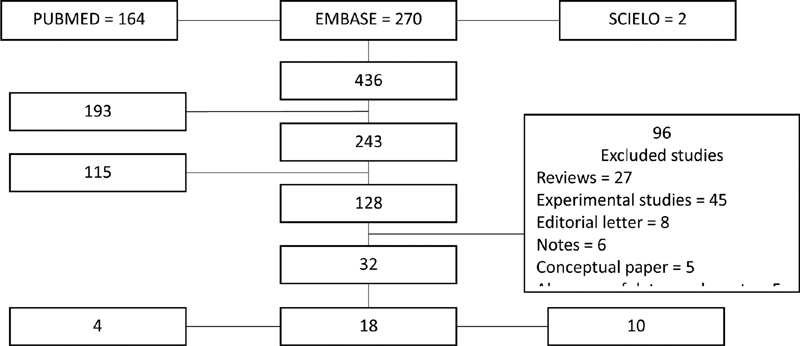
Inclusion flowchart of studies in the present review.

A total of 436 articles were retrieved in the databases search (PubMed = 164; EMBASE = 270 and SCIELO = 2); of those, 193 were duplicated articles, so 243 had their title screened. One hundred and fifteen articles were excluded after title screening, and the remaining 128 studies had their abstract read. After that, 96 studies were excluded (27 reviews, 45 experimental studies, 8 editorials, 6 notes, 5 conceptual articles, and 5 articles with no data on placental findings), and 32 studies were included in the final analysis: 18 case reports, 10 case series, and 4 cohort studies. Fig. 1 shows the inclusion flowchart for the present study.

**Fig. 2:**
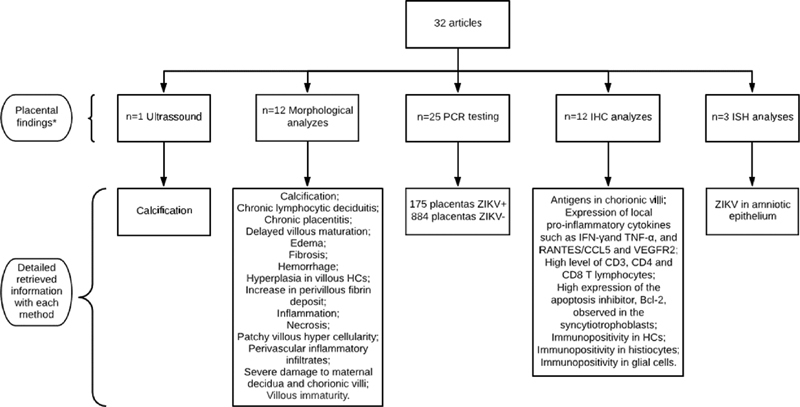
Inclusion flowchart of studies.

The majority of studies included placental testing for ZIKV with RT-PCR as part of diagnostic procedures, and some studies presented detailed data on abnormal morphological findings and immunohistochemistry (IHC) studies ( Fig. 2 ).

Table 1 summarizes the main findings on the selected studies, published from 2016 (first reports on the subject) to June 2019, containing the results of 1,244 women with ZIKV infection during pregnancy. The majority of women presented symptoms in the first trimester of pregnancy. Different methods for ZIKV infection diagnosis were performed. Placental pathologic findings were described as mild and nonspecific, including chronic placentitis (TORCH type), chronic villitis, increased HCs, variable perivillous fibrin and mononuclear cells, villous immaturity, stromal fibrosis and calcification, increased vascularity, and also lymphocytic deciduitis and focal syncytiotrophoblast necrosis. 10 11 12 13

**Table 1:** Characteristics, number of cases, and main findings on placental evaluation of the included studies

Author, year	Type of study	Participants/placentas sampled	Onset of symptoms (trimester)				Perinatal outcomes				Main findings on placental evaluation and overall findings
			1 ^st^	2 ^nd^	3 ^rd^	N/A	A	B	C	D	
Driggers et al (2016) 14	Case report	1/1	1	–	–	–	–	–	1	–	High viral load found in placenta, fetal membranes and umbilical cord by RT-PCR. ZIKV RNA in Amniotic fluid, fetal brain, liver, lung and spleen.
Martines et al (2016) 11	Case report	4 / 4	2	–	2	–	2	–	–	2	Placenta with fibrosis, calcification, and deposits of fibrin. Material consistent with third trimester gestation = RT-PCR negative. Two abortions, one had dense and heterogeneous chorionic villitis with calcification, sclerosis, edema, increased perivillous fibrin deposition, and patchy lympho histiocytic intervillositis and the other had minute fragments of placental. Both placental RT-PCR positive for ZIKV.
Martines et al (2016) 12	Case series	5/2	1	–	–	4	2	–	2	1	RT-PCR ZIKV positive in all samples. IHC: ZIKV antigens in chorionic villi of a first trimester placenta.
Melo et al (2016) 15	Case series	11/11	9	1	–	1	–	–	3	8	PCR ZIKV performed in nine placentas, two positives.
Mlakar et al (2016) 16	Case report	1/1	1	–	–	–	–	1	–	–	Ultrasound scan performed at 29 weeks showed microcephaly with brain and placental calcification. RT-PCR ZIKV positive in fetal brain tissue
Noronha et al (2016) 13	Case report	5/3	3	–	1	1	1	–	–	4	RT-PCR ZIKV positive in all samples. Main pathological findings: chronic placentitis, hyperplasia in villous HCs. IHC with the 4G2 anti-flavivirus monoclonal antibody analysis showed: immunopositivity in HCs and some histiocytes in intervillous spaces, diffusely distributed immunopositivity in some glial cells.
Sarno et al (2016) 17	Case report	1/1	–	–	–	–	–	–	1	–	RT-PCR ZIKV negative in placental sample.
van der Eijk et al (2016) 18	Case report	1/1	1	–	–	–	1	–	–	–	Placental histopathological and IHC investigation: no inflammation markers. RT-PCR ZIKV positive in the amniotic fluid, fetal and placental tissue. In situ hybridization (ISH) only found ZIKV in amniotic epithelium.
Acosta-Reyes et al (2017) 19	Case report	2/2	1	1	–	–	1	–	–	–	Placental ZIKV RT-PCR positive in one case. Histological analysis: increase in perivillous fibrin deposit, chronic lymphocytic deciduitis in both cases.
Bhatnagar et al (2017) 20	Case series	44/44 ^H^	19	24	–	1	11	3	3	27	ZIKV RT-PCR positive in 32 placental samples. ISH positive in 16 of the cases positives by RT-PCR.
Chen et al (2017) 21	Case report	1/1	–	1	–	–	–	–	–	1	RT-PCR ZIKV negative in placental sample.
Mattar et al (2017) 22	Case report	1/1	–	1	–	–	–	–	–	1	RT-PCR ZIKV positive in placental sample. ZIKV not found in umbilical cord or serum.
Rabelo et al (2017) 23	Case report	1/1	–	–	–	1	–	–	–	1	IHC revealed presence of ZIKV antigens. Severe damage to maternal decidua and chorionic villitis, with large areas of fibrinoid necrosis and perivascular inflammatory infiltrates. Dense and heterogenous calcification observed.
Reagan-Steiner et al (2017) 24	Case series	627/627	131	153	–	343	81	–	–	546	Of 546, 60 placental sample RT-PCR ZIKV positives. In 81 samples of pregnancy losses, 18 placental sample RT-PCR ZIKV positives. IHC performed in 91 placentas from livebirths and 7 had evidences of ZIKV infection.
Ritter et al (2017) 10	Case report ^E^	4/2	2	–	–	2	1	–	1	– ^F^	One sample, histological analysis showed: patchy villous hypercellularity, focal perivillous fibrin deposition, increased HCs and focal calcification.
Rosenberg et al (2017) 9	Case report	1/1	1	–	–	–	–	1	–	–	RT-PCR ZIKV positive in the placenta and fetal brain. Placenta demonstrated focally stromal edema, hydropic chorionic villi with hyperplasia and focal proliferation of HCs. prominent hypercellularity of the villous stroma. IHC with inflammatory markers (CD163 and CD8) found in HCs. ISH positive for ZIKV demonstrated scattered, strongly positive staining cells within the villous stroma of the chorionic villi, which were presumably HCs.
Schaub et al (2017) 25	Case series	8/8	6 ^I^	–	–	–	3	–	–	1 ^F^	RT-PCR ZIKV positive in three samples.
Schwartz (2017) 26	Case series	12/12	–	–	–	12	–	–	–	12	Placentas from fetuses with congenital ZIKV infection didn't present placental inflammation. IHC: special stains reveal proliferation and prominent hyperplasia of HCs, in the chorionic villi of infected placentas. ZIKV infection present in HCs from second and third trimester placentas.
Noronha et al (2018) 27	Case series	24/24	5	8	6	5	1	–	–	23	Villous immaturity was the main histological finding. IHC: Hyperplasia of HCs observed in the third trimester in placental tissues. HCs were the only ZIKV-positive fetal cells found in placentas that persisted until birth. 33% of women infected during pregnancy gave birth to babies with congenital anomalies. No pattern correlating the gestational stage in the infection, the positivity of HCs in the placenta due to IHC and the presence of congenital malformations at birth.
Esquivel et al (2018) 28	Cohort	3/6	1	2	1	–	–	–	–	3	Patient 1: Placental PCR negative, but both twins were PCR-positive. Patient 2: Both placentas and twins were PCR-positive at birth. Patient 3: One twin and associated placenta PCR-positive, the second twin and placenta PCR-negative. All six placentas with villous immaturity and other placental histopathologic findings distinct in each placental pair. These results suggest that each twin should be evaluated individually for Zika infection as ZIKV may not transmit equally to each fetus.
Maykin et al (2018) 29	Cohort	29/29	2	22	1	4	–	–	–	29	PCR ZIKV positive in 25 placentas. Ten placental pathological findings: delayed villous maturation, chronic deciduitis, stromal fibrosis and HC hyperplasia.
Mletzko e Schildgen (2018) 30	Cohort	301/121	–	–	–	300	180	–	–	121	RT-PCR ZIKV negative in all placentas and tissues from spontaneous abortions.
Rabelo et al (2018) 31	Case report	1/1	1	–	–	–	1	–	–	–	Histological analyses of the placenta and fetal organs revealed different types of tissue abnormalities: inflammation, hemorrhage, edema and necrosis in placenta, as well as tissue disorganization in the fetus. IHC: Increased cellularity (HCs and TCD8+ lymphocytes), expression of local pro-inflammatory cytokines such as IFN-γ and TNF-α, and other markers, such as RANTES/CCL5 and VEGFR2, supported placental inflammation and dysfunction.
Sassetti et al (2018) 32	Case report	1/1	1	–	–	–	–	–	–	1	RT-PCR ZIKV negative in Placenta and blood. RT-PCR positive in the newborn urine sample collected on day 1 after birth.
Turley et al (2018) 33	Case series	4/4	–	–	–	4	–	–	–	4	All 4 cases demonstrated positive placental testing by multiple modalities. IHC: tissues were stained against E glycoprotein of the ZIKV envelope with 4G2 monoclonal antibody revealing strong but localized signal in the chorionic villus parenchyma and villous lumen. PCR amplicons of the ZIKV genome were amplified by RT-PCR from placenta in all cases. Bioanalyzer assessment of RT-PCR product confirmed the highest amounts of ZIKV in placenta and verified amplicon size.
Wongsurawat et al (2018) 34	Case report	1/1	1	–	–	–	1	–	–	–	PCR ZIKV positive in the placenta and brain.
Felix et al (2017) 35	Case report	2/2	2	–	–	–	–	–	–	2	PCR ZIKV negative in placenta fragments, blood and urine. ZIKV serology performed only showed presence of IgG antibodies of maternal origin.
Merriam et al (2019) 36	Cohort	70/70	–	–	–	70	–	4	–	63	PCR positive in 1 placenta. PCR positive in urine and serum in five and nine women respectively, and two women had both positive urine and serum PCR.
Rodó et al (2019) 37	Case series	72/72	16	41	14	–	–	–	–	–	RT-PCR ZIKV positive for 10 placentas. Sorological assay positive for the other 62 women. 2 cases of central nervous system anomalies and 1 miscarriage, all in women with first trimester infection.
Santos et al (2020) 38	Case report	1/1	1	–	–	–	–	–	–	1	Deciduitis present on maternal surface of the placenta, with a prevalence of lymphocytes associated with vasculitis. IHC: HCs found in placental tissue, specific-ZIKV protein found in placental cells. IHC with high level of CD3 T lymphocytes present in addition to CD4 and CD8 cells. High expression of the apoptosis inhibitor, Bcl-2, observed in the syncytiotrophoblasts.
Seferovic et al (2019) 39	Case series	4/4	2	2	–	–	–	–	–	4	RT-PCR ZIKV performed on placenta, membrane and cord samples. ZIKV was detected in all placental specimens in cases 1, 2 and 3 (affected by CZS), but not by case 4 (unaffected). ZIKV detected in the membranes and cord of cases 2 and 3, but not in cases 1 and 4. Histological and IHC examination of placentas reveals evidence of ZIKV infection and active in cases 1, 2 and 3. ISH positive in cases 1, 2 and 3.
Yarrington et al (2019) 40	Case report	1/1	–	–	–	–	–	–	–	1	RT-PCR ZIKV positive in placental sample. Evaluation for other causes of microcephaly negative.

Abbreviations: Bcl-2, B-cell lymphoma-2; CZS, congenital Zika syndrome; HCs, Hofbauer cells; IDC, immunohistochemistry; IFN-γ, interferon-gamma; ISH, in situ hybridization; RANTES, regulated on activation, normal T expressed; RNA, ribonucleic acid; RT-PCR, reverse transcription-polymerase chain reaction; TNF-α, tumor necrosis factor-alpha; VEGFR2, vascular endothelial growth factor receptor 2; ZIKV, Zika virus.

A, abortion; B, termination; C, stillbirth; D, liveborn; E, case reports + review of the literature; F - data not detailed on all considered cases; G, asymptomatic case; H - 8 cases of children with microcephaly, without placental samples; I, two cases were asymptomatic.

Most of the detailed cases represented first-trimester infection, with symptomatic disease, leading to significant cases of abortions, stillbirth, or neonatal death. 9 10 11 12 13 18 19 20 21 25 The ZIKV was found to induce fetal disease and/or adverse pregnancy outcomes well beyond the first trimester, even late during pregnancy. 41 42

Among the reported studies, the largest case series considered 15 focused on ZIKV-specific RT-PCR amplification products from placenta with no details on IHC findings. Nevertheless, a few studies have presented interesting IHC results, with evidence of ZIKV infection in HCs within the placental villi. 12 13 20

## Discussion

The current review evaluated studies that reported placental findings among women with ZIKV infection during pregnancy. Placental pathological findings are mostly mild and nonspecific, suggesting an important role for HCs within the villi. These findings are consistent with the effects of other viruses in the placenta, such as human CMV, 43 44 leading to proinflammatory responses, impaired remodeling of spiral arteries in the decidua, and cell death; ultimately compromising suitable utero-placental blood-flow. 45 The amount of placental inflammation is associated to the severity of fetal findings. 46

The present review points toward an important role of HCs, which are of fetal origin, monocytic derived, and part of the normal component of the stroma of the chorionic villi, shown to appear very early in gestation. Hofbauer cells have been described as alternatively activated macrophages 9 47 responsible for the phagocytosis of fluids and apoptotic materials, antigen presentation, and perhaps an angiogenic role in early placental vasculogenesis, placental water balance, and endocrine function. Hyperplasia of the HCs has been previously reported in other maternal-fetal infections, such as those in the TORCH group and its proliferation within the chorionic villous stroma is also confirmed. 9 48 49

The placenta is an important virus reservoir, that can confirm the diagnosis when infection was not confirmed during the acute phase, due to limitations on adequate and timely sample collection, which is a serious concern in ZIKV infection. 4

There is a worldwide variation regarding antenatal screening availability and follow-up for women with fetal congenital abnormalities. In Latin America, many countries, including Brazil, consider abortion or termination of pregnancy due to fetal congenital abnormalities illegal or highly restricted. 50 Both factors help explain the sparsity of tissue samples from earlier gestational ages reported in the literature. A possible bias from our results is that the placental tissues evaluated were from late-pregnancy infection or infections in apparently unaffected neonates. 50

Another important point our review highlights is that there is no standardized description of placental findings related to ZIKV. A common global pattern of description of those findings would be helpful to gather results from different groups, settings and countries, allowing researchers to empower results and provide more robust conclusions. It would also help clinicians to justify the importance of histological analysis of placental tissue in suspect or confirmed cases of ZIKV during pregnancy.

## Conclusion

Characterizing placental infection is key for understanding the severity of the disease and fetal malformations. The ZIKV presents similar features to other TORCH infections, with a significant role of HCs. Missed opportunities of such evaluation are evident when considering the limited number of studies included in the present review. However, it is very important to address the need for adequate sampling and evaluation of placental findings during an outbreak, among suspected and confirmed cases of ZIKV infection. For that, specific evaluation on different placental layers, combined with studies on RNA detection and standardization of results presentation is fundamental.
